# Assessment of image co-registration accuracy for frameless gamma knife surgery

**DOI:** 10.1371/journal.pone.0193809

**Published:** 2018-03-02

**Authors:** Hyun-Tai Chung, Jeong Hun Kim, Jin Wook Kim, Sun Ha Paek, Dong Gyu Kim, Kook Jin Chun, Tae Hoon Kim, Yong Kyun Kim

**Affiliations:** 1 Department of Neurosurgery, Seoul National University College of Medicine, Seoul, Korea; 2 Department of Neurosurgery, Seoul National University Hospital, Seoul, Korea; 3 Department of Accelerator Science, Korea University Sejong Campus, Sejong, Korea; 4 Department of Nuclear Engineering, Hanyang University College of Engineering, Seoul, Korea; University Health Network and University of Toronto, CANADA

## Abstract

Image co-registration is used in frameless gamma knife radiosurgery (GKSRS) to assign a stereotactic coordinate system and verify patient setup before irradiation. The accuracy of co-registration with cone beam computed tomography (CBCT) images of a Gamma Knife Icon^TM^ (GK Icon) was assessed, and the effects of the region of co-registration (ROC) were studied. CBCT-to-CBCT co-registration is used for patient setup verification, and its accuracy was examined by co-registering CBCT images taken at various configurations with a reference CBCT series. The accuracy of stereotactic coordinate assignment was investigated by co-registering stereotactic CT images with CBCT images taken at various configurations. An anthropomorphic phantom was used, and the coordinates of fifteen landmarks inside the phantom were measured. The co-registration accuracy between stereotactic magnetic resonance (MR) and CBCT images was evaluated using images from forty-one patients. The positions of the anterior and posterior commissures were measured in both a fiducial marker-based system and a co-registered system. To assess the effects of MR image distortions, co-registration was performed with four different ranges, and the accuracy of the results was compared. Co-registration between CBCT images gave a mean three-dimensional deviation of 0.2 ± 0.1 mm. The co-registration of stereotactic CT images with CBCT images produced a mean deviation of 0.5 ± 0.2 mm. The co-registration of MR images with CBCT images resulted in the smallest three-dimensional difference (0.8 ± 0.3 mm) when a co-registration region covering the skull base area was applied. The image co-registration errors in frameless GKSRS were similar to the imaging errors of frame-based GKSRS. The lower portion of the patient’s head, including the base of the skull, is recommended for the ROC.

## Introduction

Before the Gamma Knife Icon^TM^ (GK Icon) was introduced in 2015, most gamma knife radiosurgeries (GKSRSs) were performed with a stereotactic frame. This frame immobilized the patient’s head during stereotactic procedures and generated a stereotactic coordinate system so that the location of the target could be accurately determined [1]. Although a frame provides sub-millimeter accuracy for target localization with an appropriate imaging tool [2,3], the frameless methods were devised mainly to fractionate intracranial treatments in other stereotactic radiosurgery modalities, such as CyberKnife^®^ and other linac-based procedures. The GK evolved simultaneously, and frameless GKSRS became available when cone beam computed tomography (CBCT) and a high-definition motion management system were combined to produce the GK Icon. The CBCT has two roles in frameless GKSRS. First, the CBCT assigns a stereotactic coordinate system by co-registering clinical magnetic resonance (MR) and/or CT images to a reference CBCT image series. Second, the CBCT verifies the patient setup before irradiation by co-registering a CBCT image series with a previously taken reference CBCT series. Patient immobilization during irradiation is accomplished with a mask and the high-definition motion management system. The accuracy of image co-registration must be assessed because irradiation is administered according to the coordinate system and patient setup established based on co-registration.

Before the GK Icon, accurate evaluation of the co-registration between clinical serial CT and/or MR images and CT images was required only to determine the accuracy of target delineation or pre-planning. The co-registration accuracy between CT and MR images using old versions of GKSRS treatment planning software (Leksell Gamma Plan; LGP, Elekta AB, Stockholm, Sweden) were evaluated by Watanabe and Han for LGP version 8.0 [4] and by Nakazawa et al. for LGP version 10.1.1 [5]. These authors reported co-registration errors of less than 1.2 mm. However, the accuracy of co-registration using the CBCT images of a GK Icon should be independently examined because CBCT generates intrinsic images with characteristics that differ from those of other ordinary CT images. The fact that a different version of LGP, version 11.0, is used in the GK Icon is another reason to perform separate accuracy evaluations. LGP version 11.0 uses Normalized Mutual Information (NMI) to co-register images. The NMI is computed using histogram data and a stochastic sampling of the image intensities using linear partial volume interpolation [6]. The manufacturer of the GK Icon announced in a white paper that its co-registration errors were less than 0.6 mm between synthetically generated CBCT images and the MR images of five patients [6]. This accuracy, however, should be carefully verified using actual CBCT images and clinical serial CT and/or MR images. Recently, Li et al. reported that the mean interfraction patient setup errors of frame-based GKSRS were less than 0.5 mm and that the intrafraction errors were approximately 0.03 mm [7]. The authors used LGP version 10.1 and a research prototype CBCT, which was attached to an existing GK Perfexion. Their results on interfraction and intrafraction patient movements can be re-evaluated based on the co-registration accuracies of the current study.

In this study, the accuracy of co-registration procedures with various imaging modalities was thoroughly assessed. The accuracy of the co-registration between a reference CBCT series and other CBCT images was investigated using an anthropomorphic phantom in various positions. The accuracy of the stereotactic coordinate system assignment was determined by measuring the co-registration errors between clinical serial CT images and CBCT images of the phantom. Co-registrations between stereotactic MR images and CBCT images were examined using patient images.

## Materials and methods

### CBCT of the GK Icon

**Fig 1:**
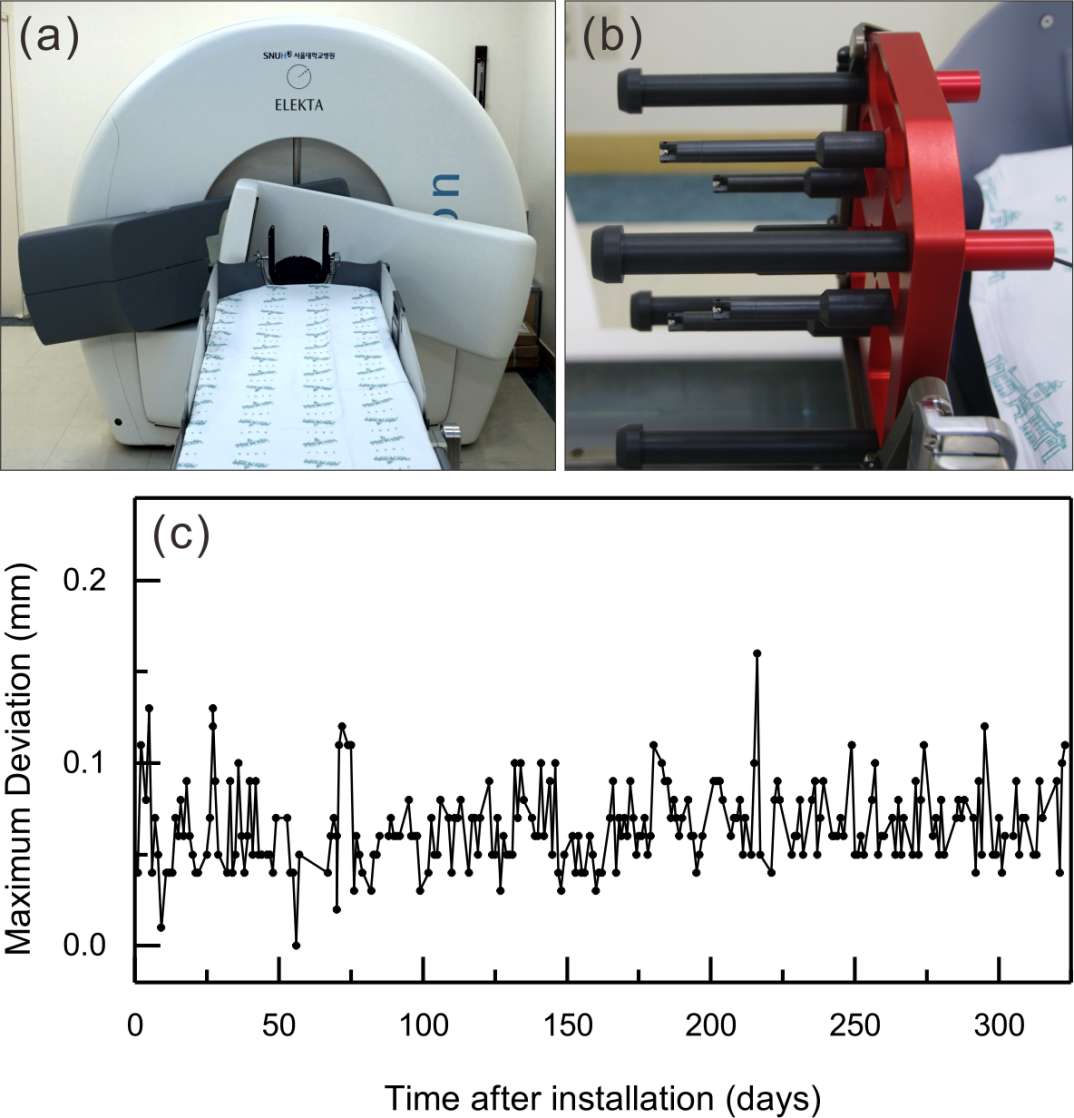
The cone beam computed tomography (CBCT) of a Gamma Knife Icon. (a) The CBCT at its maximum rotation angle for imaging. (b) A CBCT geometrical accuracy verification phantom. There are four ball bearings with known positions. (c) The maximum deviations of the four ball bearings were measured throughout the year after installation.

Fig 1A shows the CBCT of the GK Icon located at its maximum rotation angle. The setup was designed to produce tomographic images with a rotation angle of 200° because the C-arm cannot pass through the patient couch. The source-to-axis distance was 790 mm, and the source-to-detector distance was 1000 mm. The cone beam angle was 15°, and the fan angle was 16°. The reconstructed volume was 224 x 224 x 224 mm^3^, and the voxel size of an image was 0.5 x 0.5 x 0.5 mm^3^. The CBCT image slice thickness was 0.5 mm. The CBCT imaging parameters and their weighted CT dose index (CTDI_w_) values were provided by the manufacturer. Two presets produce CTDI_w_ values equal to 2.5 mGy and 6.3 mGy, respectively. The operating peak voltage was 90 kVp. The operating current was 0.4 mA/projection for CTDI_w_ = 2.5 mGy and 1.0 mA/projection for CTDI_w_ = 6.3 mGy. The geometrical accuracy of the CBCT images was measured with a vendor-provided phantom (Fig 1B). The positions of the four ball bearings of the phantom were measured in CBCT images, and the maximum three-dimensional deviation was used as the geometrical error. Fig 1C shows the daily fluctuations of the geometrical error for one year after installation; the mean value was 0.07 ± 0.02 mm.

### Evaluation of co-registration errors

**Fig 2:**
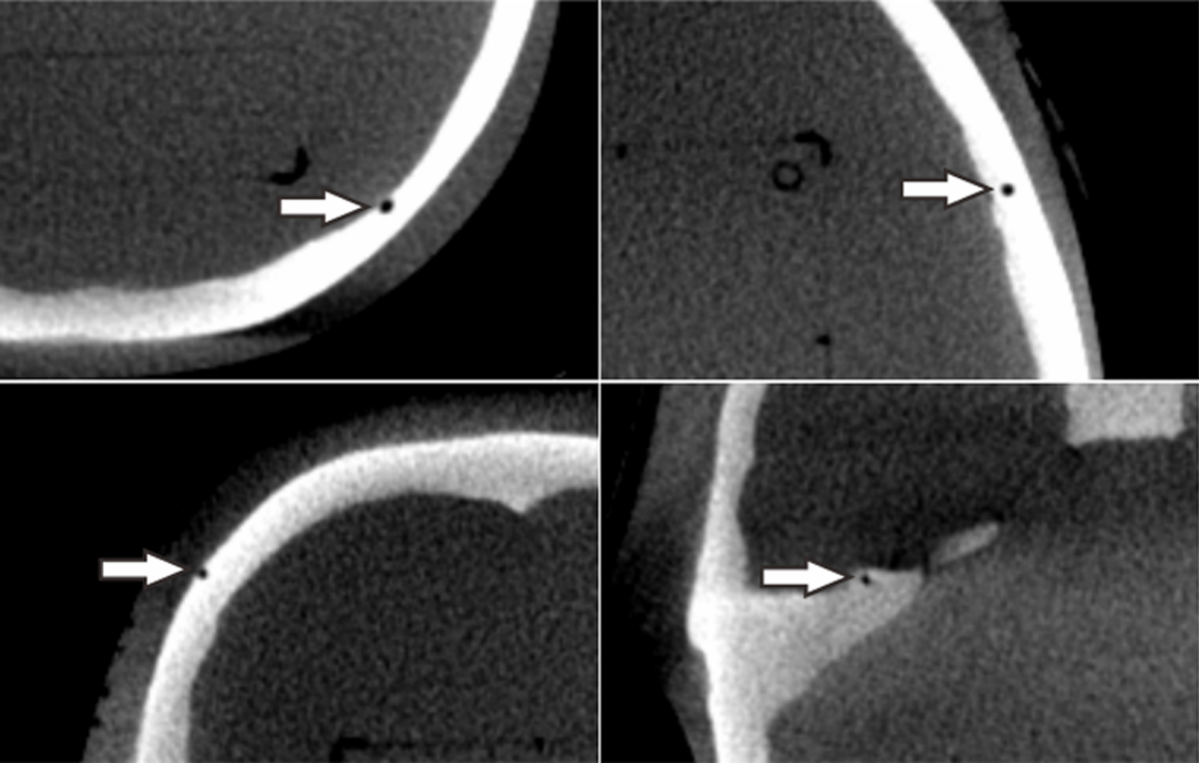
Examples of the landmarks. The white arrows show four of the fifteen arbitrary landmarks used for coordinate measurements in a reference CBCT image set of a CIRS radiosurgery head phantom.

The co-registration error along an axis was defined as the mean one-dimensional deviation of the fifteen landmarks found in a commercial anthropomorphic phantom (CIRS Radiosurgery Head Phantom Model 605, CIRS Inc., Norfolk, VA, USA) (Fig 2). The landmarks, air bubbles and impurities in the phantom were chosen arbitrarily so that their maximum coordinate values had a distribution similar to the geometrical limits of frame-based GKSRS. The center of the CBCT-based coordinate system was (100.0, 100.0, 100.0), and the coordinate values of the landmarks ranged from 47.3 mm to 160.3 mm, from 21.6 mm to 172.4 mm, and from 3.9 mm to 160.4 mm along the x-axis (right to left), y-axis (posterior to anterior), and z-axis (head to feet), respectively. The coordinate values of each landmark were measured independently four times by two physicists. One-dimensional deviations were obtained by subtracting the coordinate values of a point measured in the co-registered images from the coordinate values of the same landmark measured in the reference images. The three-dimensional deviation was defined as the root mean square value of the three one-dimensional deviations.

### Accuracy of co-registration between CBCT images

In a clinical situation, the movement between each patient setup was corrected by adjusting the couch movements according to the deviations found in the co-registration of a CBCT image set with a reference CBCT set. Image co-registration was performed using the GK treatment planning software LGP version 11.0. To evaluate the accuracy of this co-registration, the phantom was fixed to the patient couch using a mask, and then a reference CBCT image set with CTDI_w_ = 6.3 mGy (CBCT_A1) was taken. Without moving the phantom, two more CBCT sets were taken with CTDI_w_ = 6.3 mGy, and an additional two CBCT sets were taken with CTDI_w_ = 2.5 mGy to examine the differences between the two CBCT imaging protocols. Then, without any movement, CBCT images were taken five times using a CTDI_w_ = 2.5 mGy protocol to check the repeatability of co-registration. CBCT images were then taken after the mask was removed, and the phantom was rotated around the z-axis up to 10° clockwise and counter-clockwise in 2° steps. Finally, the phantom was placed in five positions with arbitrary translations and rotations. The deviation of the center of the moved image sets ranged from 24.07 mm to 36.58 mm; thus, the movements were large enough to cover most ordinary clinical cases. The CTDI_w_ = 2.5 mGy protocol was applied for imaging in the rotated or moved positions because this protocol is commonly used in patient setup verifications. The coordinate values of the fifteen landmarks measured in the CBCT image sets co-registered with CBCT_A1 were compared to those determined in CBCT_A1.

### Accuracy of co-registration between CBCT and CT images

**Fig 3:**
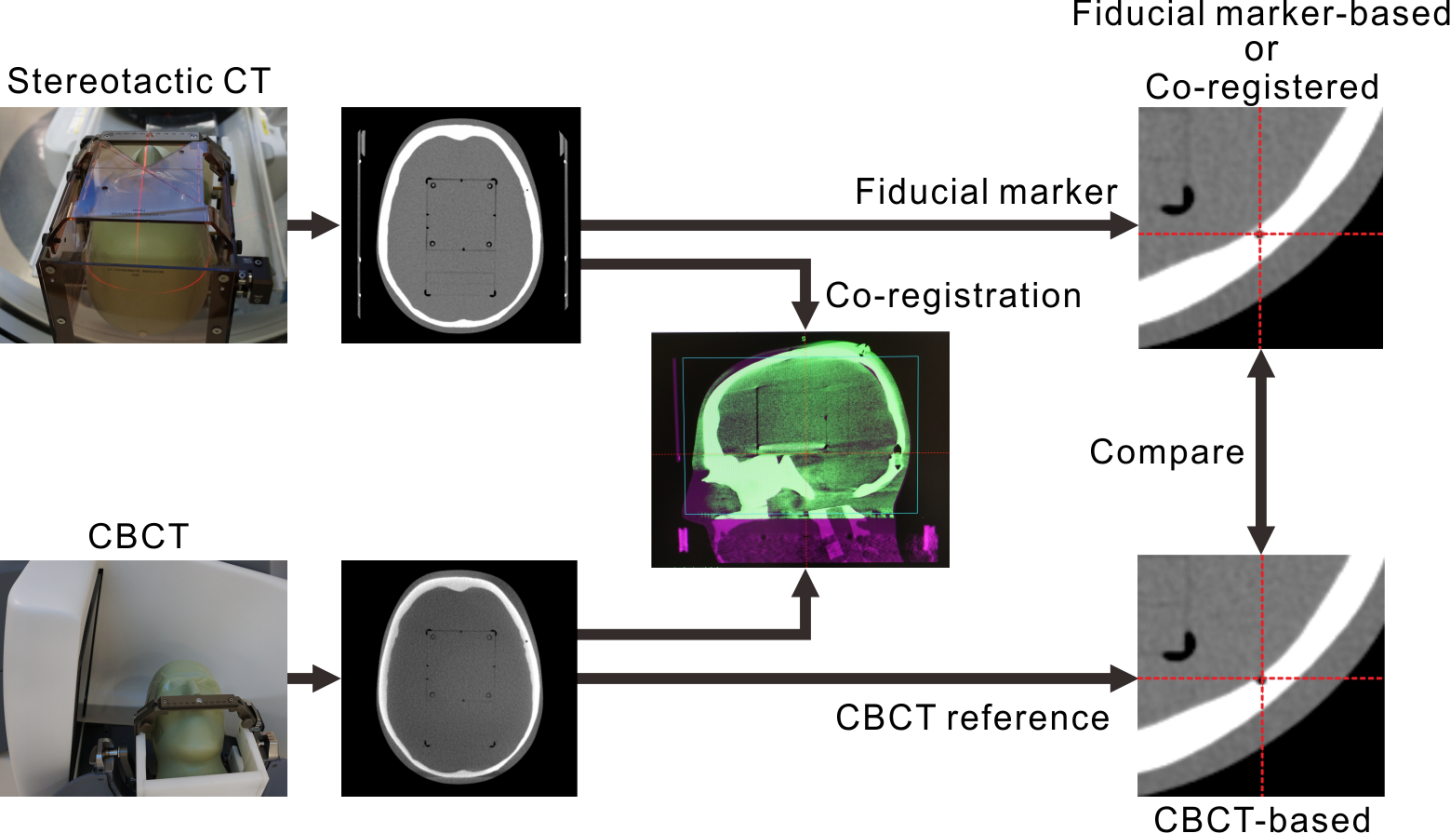
The procedure used to examine the accuracy of stereotactic coordinate assignment. A fiducial marker-based system was defined using the fiducial markers in the stereotactic CT images. A co-registration-based system was assigned by co-registering the stereotactic CT images with reference CBCT images. A CBCT-based system was intrinsically defined so that its center was calibrated to the radiation focus. The coordinates of the landmarks measured in each coordinate system were compared.

The stereotactic coordinate system of a frameless GKSRS is defined by a reference CBCT image set, and it is assigned to clinical images, such as ordinary CT images, by co-registering these images with the reference. The accuracy of the stereotactic coordinate assignment procedure was investigated by comparing the coordinate values measured in the co-registered CT images to those obtained from the reference CBCT images. The CIRS 605 phantom was combined with a Leksell stereotactic G-frame (Elekta AB, Stockholm, Sweden) using a homemade device, and its stereotactic CT images (CT_B1, CT_B2) were taken using a GE Discovery^TM^ CT750 HD (GE Medical Systems Inc., Waukesha, WI, USA), as shown in Fig 3. A stereotactic neurosurgery protocol (i.e., 120 kVp, 250 mA, 1.25-mm thickness, and 0.49-mm pixel size) was applied in helical mode for imaging. A fiducial marker-based coordinate system was assigned to the images using the fiducial markers produced by the N-shaped copper flat wires in a CT indicator (Elekta AB, Stockholm, Sweden). The fifteen landmarks in the phantom were identified, and their positions were recorded four times by two individuals. Then, the phantom was fixed to a GK Icon using the frame, and CBCT images were taken with CTDI_w_ = 6.3 mGy (CBCT_B1). CT_B1 and CT_B2 were co-registered with CBCT_B1, and the landmark positions were measured in the co-registered images. The CBCT coordinate system was used as a reference system, and the one-dimensional deviation was obtained by subtracting the co-registered coordinate from the CBCT coordinate. To simulate clinical situations, the phantom was placed in six arbitrary positions, and CBCT images were taken with CTDI_w_ = 6.3 mGy. CT_B1 was co-registered with each of the CBCT image sets. Deviations of the landmark positions in the co-registered images were measured using each CBCT image set as a reference.

### Accuracy of co-registration between CBCT and MR images

**Fig 4:**
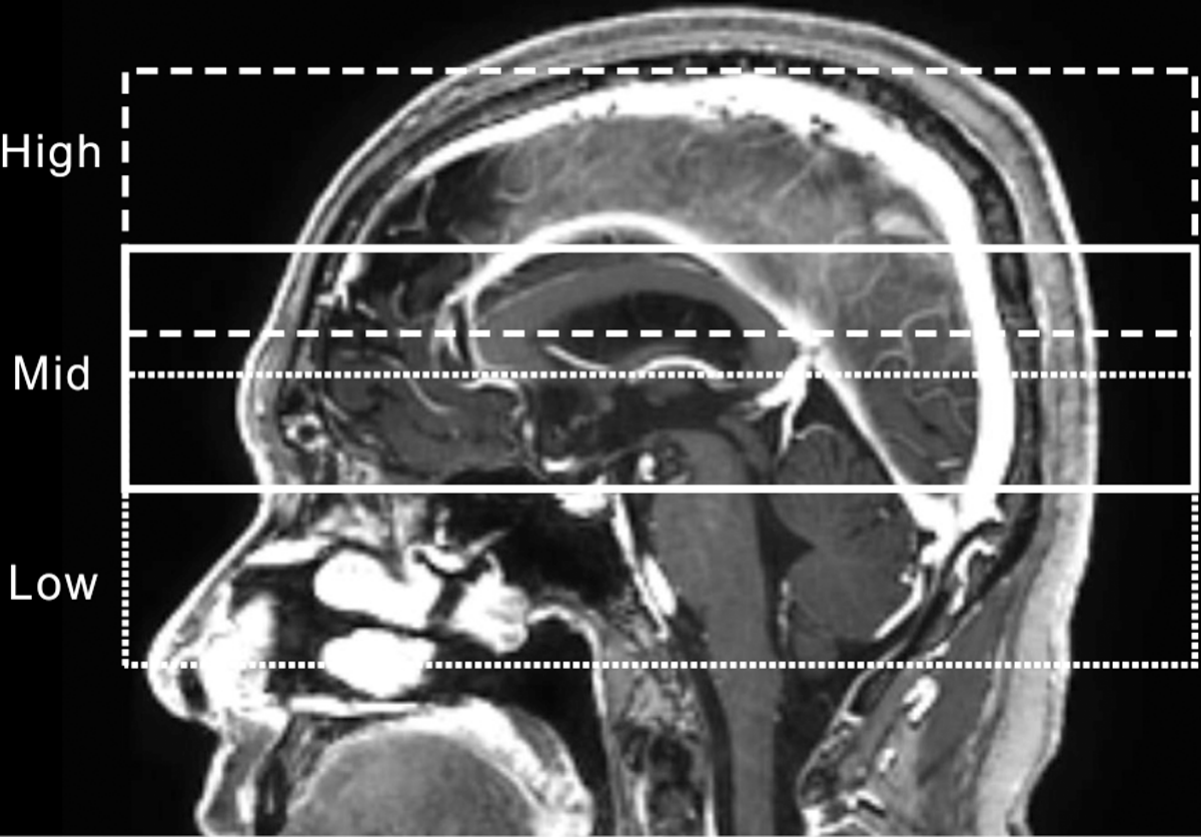
Region of co-registrations used in this study. The dotted line corresponds to the ‘Low’ region, the solid line to the ‘Mid’ region, and the dashed line to the ‘High’ region. The ‘Large’ region is the total area of all three regions.

The accuracy of stereotactic coordinate assignment based on the co-registration of stereotactic MR images with a reference CBCT image set was investigated using stereotactic MR images and CBCT images of patients treated with GK Icon from July 2016 to September 2016. Forty-one patients who were treated via frame-based GKSRS and provided informed consent for additional reference CBCT imaging with CTDT_w_ = 6.3 mGy were included. There was no informed consent from the patients to be included in this study because this was a retrospective study using anonymously analyzed imaging data without any personal information. This study including the consent procedure was approved by the IRB of Seoul National University College of Medicine (IRB No. 1611-044-806). Stereotactic MR images were taken using a GE Signa HDxT 1.5-T MR instrument (GE Medical Systems Inc., Waukesha, WI, USA). T1-weighted axial images in a multi-planar gradient recall sequence with a 0.5 x 0.5 mm resolution and 1.5 mm slice thickness were obtained, and a fiducial marker-based coordinate system was defined. Distortions of the stereotactic MR images of a commercial phantom (CIRS model 603A, CIRS Inc., Norfolk, VA, USA) attached to a Leksell stereotactic G-frame were measured by comparing them with the corresponding CT images. Distortions of patient MR images were checked by LGP—comparing the fiducial marker locations to their known values. The stereotactic MR images were co-registered with CBCT images, and a co-registered coordinate system was assigned. The locations of the anterior commissure (AC) and posterior commissure (PC) were identified in each coordinate system, and their positions were measured three times by two individuals. The repeatability of MR-CBCT co-registration was investigated by repeating the co-registration three times and measuring AC and PC locations for fifteen arbitrary selected patients. The effect of patient head size on image co-registration accuracy was also investigated. The length of a patient’s head was defined as the longest anterior to posterior distance measured along the cerebral falx in an axial image taken for GKSRS. The width was measured along a line perpendicular to the cerebral falx in the same axial image, and the area was measured by drawing a contour around the patient’s head in the same axial image. The height was defined as the distance from the bottom of the cerebellum to the vertex in a sagittal image, including the cerebral falx. The volume was defined as the volume of a cuboid calculated by multiplying the length, width, and height of the head. To investigate whether different choices of the region of co-registration (ROC) affected the accuracy, four ROCs were selected, as shown in Fig 4. Although it was not possible to exactly match the ROCs among all patients, the ‘Low’ region included the foramen magnum and the bottom of the corpus callosum; the ‘Mid’ region included the top of the pons to the top of the corpus callosum; the ‘High’ region included the middle of the lateral ventricle to the top of the brain; and the ‘Large’ region was the total area of all three regions. The coordinate values of the AC and PC measured in the images co-registered with different ROCs were compared. All the co-registrations mentioned before the ROC comparison were performed in the ‘Large’ region.

To evaluate the clinical importance of the co-registration accuracy, the maximum shot displacement, target coverage ratio, maximum and minimum doses to a target, and Paddick conformity index [8] were calculated by LGP and recorded for each coordinate system.

### Statistical analysis

All statistical analyses in this study were performed using the commercial statistics package IBM^®^ SPSS^®^ Statistics version 22 (IBM Corp, Armonk, NY, USA). An independent-sample t-test or one-way ANOVA was applied to compare the mean values of different groups, and the difference was determined to be statistically significant when the *p*-value was less than or equal to 0.05. The Pearson correlation test was used to investigate the relationships between two variables. All of the mean values reported in this study are presented together with one standard deviation.

## Results and discussion

### Accuracy of co-registration between CBCT images

**Fig 5:**
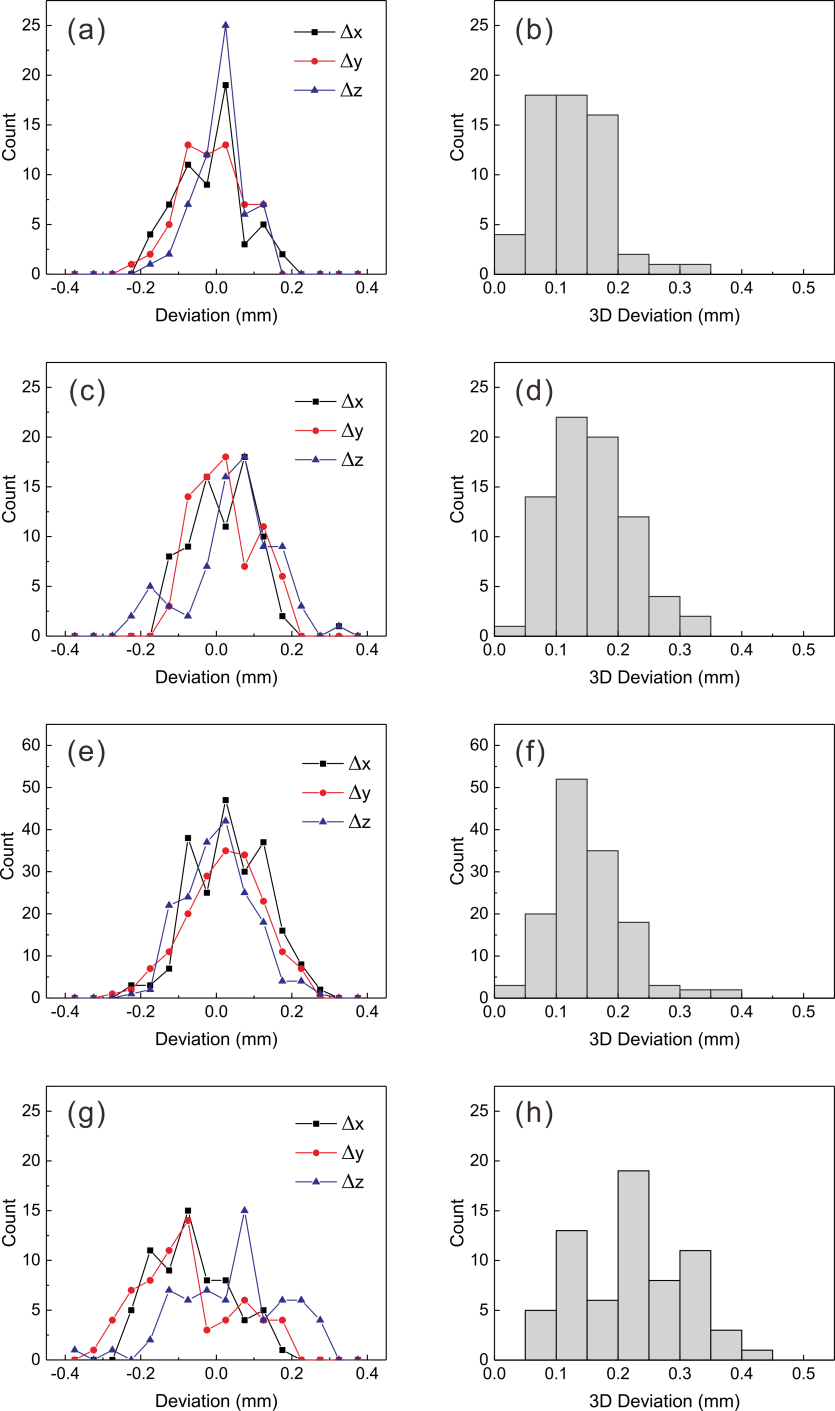
One- and three-dimensional deviations measured based on co-registration between CBCT images. (a), (b) before co-registration; (c), (d) after co-registration without any movement; (e), (f) with rotations only; and (g), (h) with arbitrary movements.

**Fig 6:**
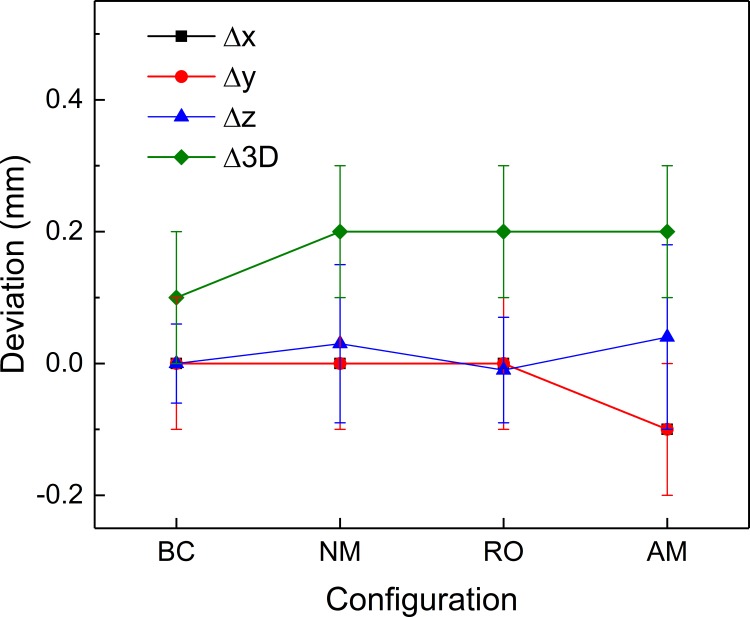
The accuracies of co-registration between CBCT images. Mean and standard deviations of the one- and three-dimensional deviations of the fifteen landmarks before and after the co-registration of CBCT images with reference CBCT images. BC: before co-registration; NM: no movement; RO: rotation only; and AM: arbitrary movement.

The coordinates of the fifteen landmarks in an image set could be measured repeatedly with a mean standard deviation of 0.1 ± 0.0 mm. The coordinates measured in CBCT images before co-registration were compared to the reference images (CBCT-A1), and the distributions of the differences are presented in Fig 5A and 5B. The mean three-dimensional difference was 0.1 ± 0.1 mm (Fig 6). No statistically significant differences were found between the CTDI_w_ = 6.3 mGy images and the 2.5 mGy images (*p* = 0.478). After co-registration of the five CBCT image sets taken without any movement, the mean three-dimensional displacement increased to 0.2 ± 0.1 mm (*p* = 0.017; Fig 5C and 5D and Fig 6). After co-registration of the CBCT images taken after rotations around the z-axis, the mean three-dimensional deviation was equivalent to that obtained using CBCT images taken without any movement (*p* = 0.384; Fig 5E and 5F and Fig 6). No dependence of the one- and three-dimensional displacements on the rotation angle up to ±10° was observed (*p* = 0.107). The mean three-dimensional displacement measured in the co-registered CBCT images taken at five arbitrary positions was 0.2 ± 0.1 mm, which was significantly different from all of the previously measured displacements (*p* = 0.000, Fig 5G and 5H and Fig 6). The five positions were statistically equivalent in terms of their three-dimensional deviations (*p* = 0.951). Although the mean Δ*x* (−0.1 ± 0.1 mm) and mean Δ*y* (−0.1 ± 0.1 mm) values were both negative and did not differ significantly (*p* = 0.523), the mean Δ*z* (0.0 ± 0.1 mm) was significantly different from Δ*x* and Δ*y* (*p* = 0.000).

### Accuracy of co-registration between CBCT and CT images

**Fig 7:**
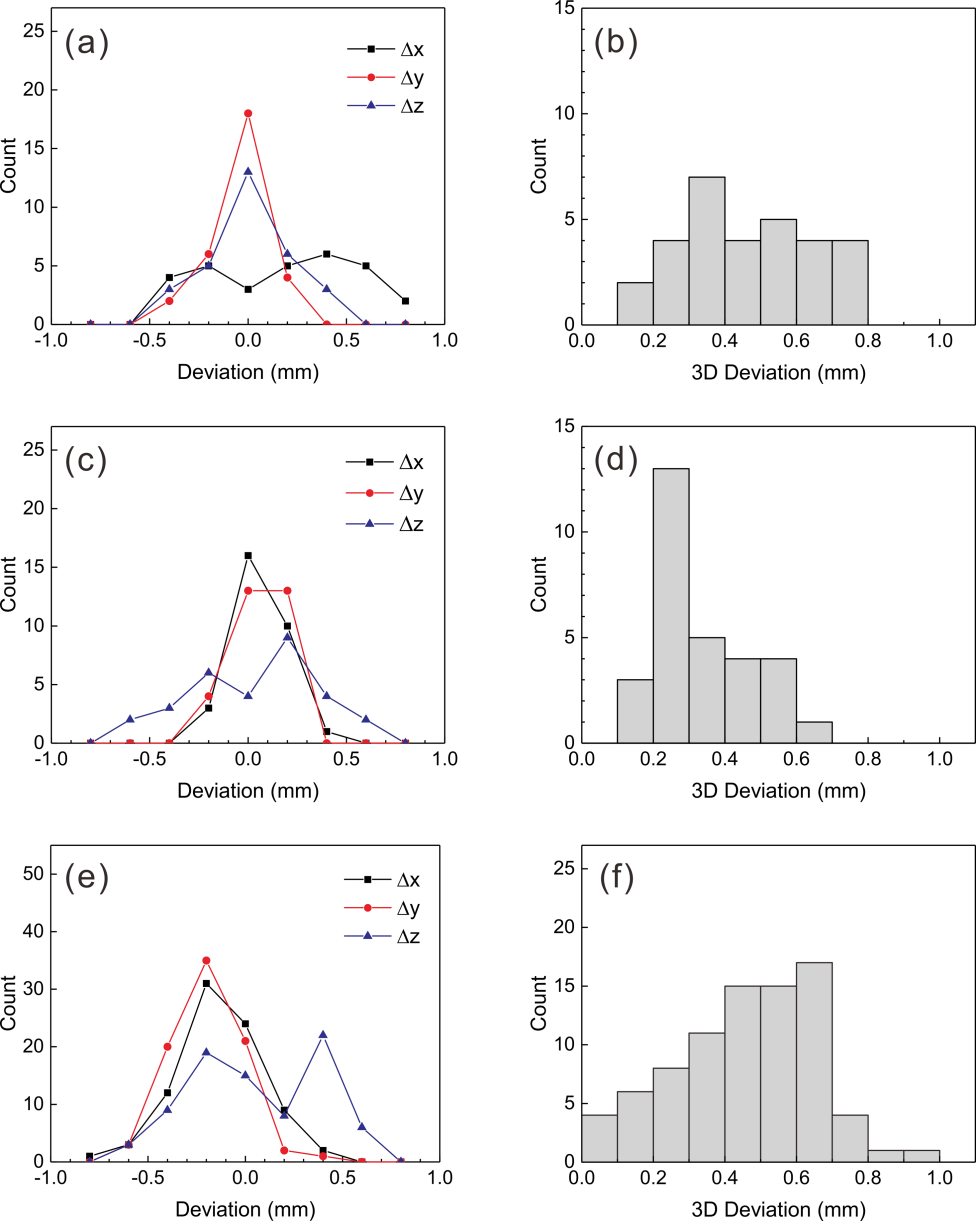
One- and three-dimensional deviations measured based on the co-registration of CBCT images and stereotactic CT images. (a), (b) before co-registration; (c), (d) after co-registration without any movement; and (e), (f) with arbitrary movements.

**Fig 8:**
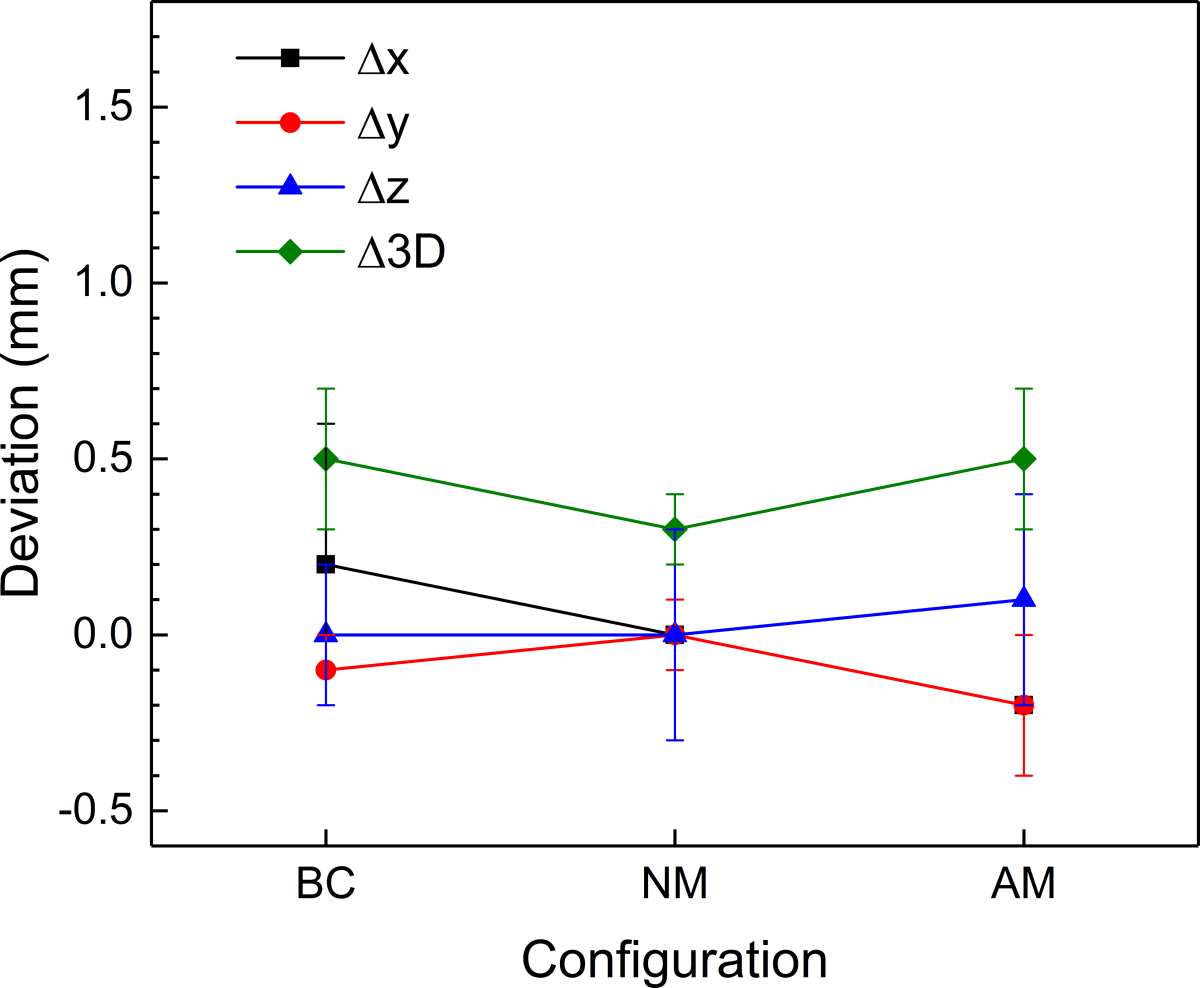
The accuracies of co-registration between CBCT and CT images. Means and standard deviations of the one- and three-dimensional deviations of the fifteen landmarks before and after co-registration between CBCT images and stereotactic CT images. BC: before co-registration; NM: no movement; and AM: arbitrary movement.

When fiducial marker-based coordinate systems were defined using CT_B1 and CT_B2, the mean error in the fiducial marker positions was 0.3 mm, and the maximum error was 0.8 mm in both image sets. The one- and three-dimensional deviations found before and after co-registration of these images with CBCT_B1 images are shown in Figs 7 and 8. Before co-registration, the mean differences in the coordinates between the fiducial marker-based system and the CBCT-based system were 0.2 ± 0.4 mm, -0.1 ± 0.1 mm, and 0.0 ± 0.2 mm along the x-, y-, and z-axes, respectively. The mean three-dimensional deviation was 0.5 ± 0.2 mm. There were no differences between CTs (CT_B1 and CT_B2, *p* = 0.609). After the stereotactic CT images were co-registered with CBCT_B1, one-dimensional deviations were 0.0 ± 0.1 mm, 0.0 ± 0.1 mm, and 0.0 ± 0.3 mm along the x-, y-, and z-axes, respectively. The three-dimensional deviation was significantly reduced to 0.3 ± 0.1 mm (*p* = 0.020). The one-dimensional deviations after co-registering the CT_B1 images with the six arbitrary position CBCT image sets were −0.2 ± 0.2 mm, −0.2 ± 0.2 mm, and 0.1 ± 0.3 mm along the x-, y-, and z-axes, respectively. The three-dimensional deviation increased to 0.5 ± 0.2 mm (*p* = 0.037) and was equivalent to the value obtained before co-registration (*p* = 0.523). The three-dimensional deviations were not dependent on the distance from the center (*p* = 0.163). Although Δ*x* and Δ*y* were both negative and were not significantly different (*p* = 0.391), Δ*z* was positive and significantly different from Δ*x* and Δ*y* (*p* = 0.002).

### Accuracy of co-registration between CBCT and MR images

**Fig 9:**
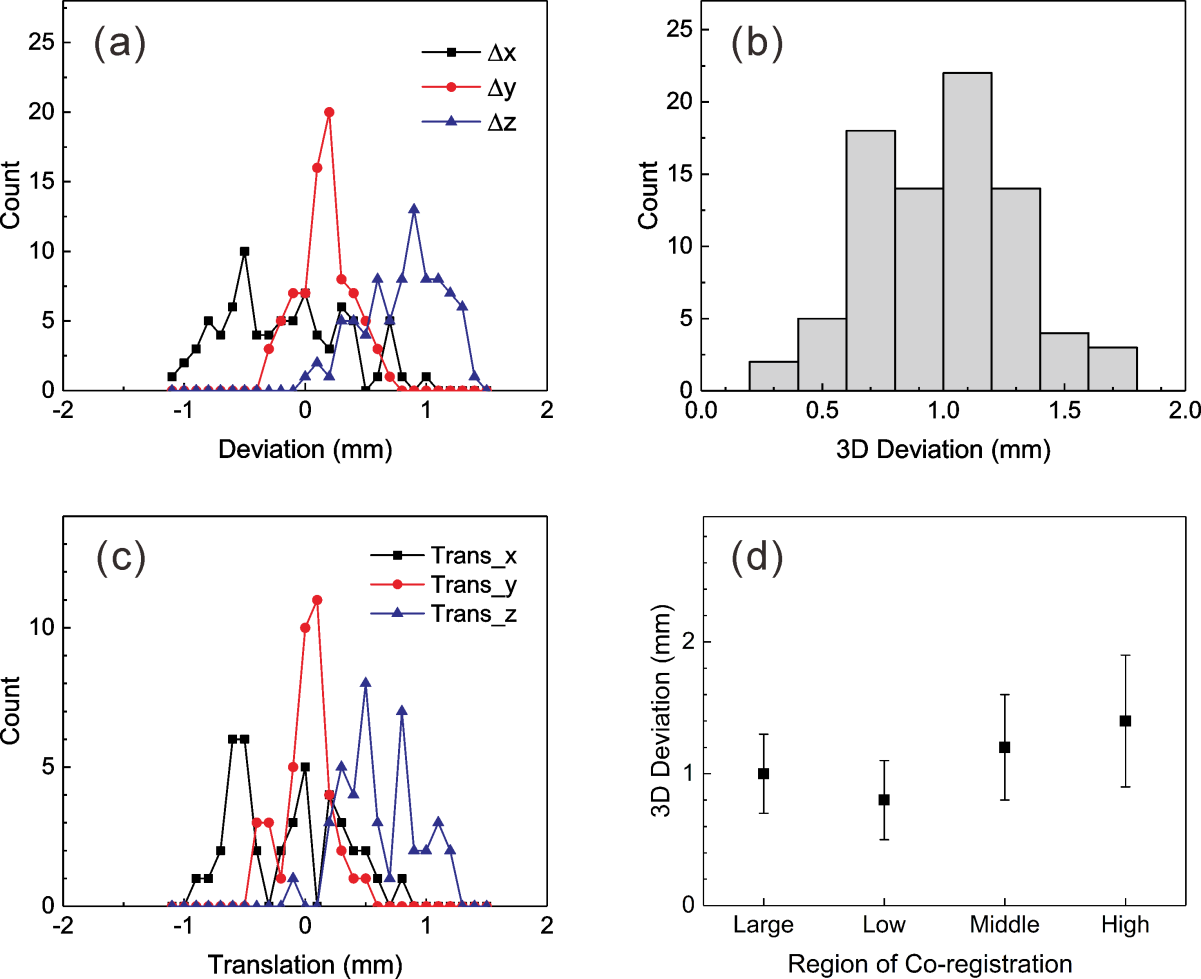
The differences between fiducial marker-based and co-registered MR images. (a), (b) One- and three-dimensional deviations of AC and PC. (c) Translation parameters reported by the co-registration program. (d) Mean three-dimensional deviations of AC and PC obtained after co-registration using four ROCs.

The mean value of MR distortions was obtained by measuring 75 points in the CIRS model 603A phantom images, and it was 0.5 ± 0.3 mm inside the phantom skull. When fiducial marker-based coordinate systems were assigned to the patient MR images, the mean fiducial marker position error was 0.5 ± 0.1 mm, and the maximum error was 0.9 ± 0.1 mm. When MR images were co-registered with corresponding CBCT images, LGP reported rotation and translation parameters based on the co-registration results. The mean values of these parameters are given in Table 1. All of these variables were supposed to be zero because the rotation of the MR indicator is corrected by LGP, and no translation was assumed [9]. The translation along the z-axis and rotation angle around the x-axis were always positive. The positions of the AC and PC were measured with a mean standard deviation of 0.1 ± 0.1 mm. The mean displacements of the AC and PC after the co-registration of MR images with CBCT images are also provided in Table 1. Both the maximum shot displacement and the mean three-dimensional displacement of the AC and PC were approximately 1.0 mm. The one-dimensional deviations exhibited similar distributions with translations along each axis, as shown in Fig 9. Because the average rotations around the y-axis and z-axis were close to zero degrees, the deviation of AC/PC and the translation of the x-axis were nearly the same. The one-dimensional deviations differed significantly from each other (*p* = 0.000), and their absolute values were close to half of the values of the MR image voxel size (0.5 x 0.5 x 1.5 mm^3^). The displacement along the z-axis was always positive.

**Table 1:** Means and standard deviations of the coordinate transformation parameters reported by LGP. One- and three-dimensional deviations in the positions of the AC and PC are also shown. (angle unit: degree, length unit: mm).

LGP		AC/PC	
Rotation(x)	0.53 ± 0.25	-	
Rotation(y)	0.07 ± 0.14	-	
Rotation(z)	0.02 ± 0.08	-	
Trans(x)	−0.16 ± 0.43	Δ*x*	−0.2 ± 0.5
Trans(y)	0.01 ± 0.20	Δ*y*	0.2 ± 0.2
Trans(z)	0.61 ± 0.33	Δ*z*	0.8 ± 0.3
Maximum shot displacement	1.00 ± 0.45	Δ3D	1.0 ± 0.3

When MR-CBCT co-registration was repeated three times for fifteen patients, the AC and PC exhibited 0.2 ± 0.1 mm three-dimensional variations in their positions. The mean anterior to posterior length of a patient’s heads was 185.5 ± 9.0 mm (range: 164.1–204.4 mm), and the mean width was 162.0 ± 6.9 mm (range: 150.0–175.5 mm). The mean ratio of the width to length was 0.87 ± 0.05, and the mean area was 237.9 ± 18.6 cm^2^. The mean height was 148.6 ± 5.3 mm, and the mean cuboid volume was 4479.5 ± 474.7 cm^3^. There was no meaningful relationship between the maximum fiducial marker position errors and any of the head size parameters. The mean fiducial marker position error showed borderline dependence on the area (*p* = 0.063) and the cuboid volume (*p* = 0.084). The co-registration accuracy assessed by three-dimensional deviations of AC and PC showed a statistically significant difference only for the ratio of the width to length (*p* = 0.045). The higher ratio showed a larger deviation.

The three-dimensional deviations of AC and PC obtained after co-registration with different ROCs are shown in Fig 9D. All four ROCs produced significantly different three-dimensional deviations (*p* ≤ 0.002), and the ‘Low’ region yielded the smallest value (0.8 ± 0.3 mm). However, no meaningful differences in the target coverage ratio (*p* = 0.218) and minimum dose (*p* = 0.519) were found between the ‘Low’ and ‘Large’ regions.

Seventy-three lesions were included in a planning parameter comparison analysis between the fiducial marker-based system and the CBCT-based system. The mean volume of the lesions was 1.95 ± 3.32 cm^3^ (median = 0.38 cm^3^), and it ranged between 0.01 cm^3^ and 18.73 cm^3^. After co-registration using the ‘Large’ region, the planning parameters automatically re-calculated by LGP were compared to previous values. Because the treatment plan was originally based on the fiducial marker-based system, the target coverage ratios in the CBCT-based system were lower: 98.5 ± 1.7% versus 90.6 ± 10.2% (*p* = 0.000). The Paddick conformity index was also smaller in the CBCT-based system: 0.65 ± 0.24 versus 0.57 ± 0.25 (*p* = 0.000). The minimum dose per target in the CBCT-based system was 78 ± 21% of that of the fiducial marker-based system (*p* = 0.000); however, there was no significant difference in the maximum dose per target (*p* = 0.642).

### Discussion

In the present study, the accuracy of co-registration using GK Icon CBCT images was assessed with anthropomorphic phantom CT/CBCT and patient MR images. The accuracy measured in this study was comparable to the values reported in previous studies showing co-registration accuracies between CT and MR images. Watanabe et al. reported the co-registration accuracy between CT and MR images of a box phantom and found mean differences of -0.97 ± 0.44 mm, 0.03 ± 0.37 mm, and 0.40 ± 0.35 for the x-, y-, and z-axes, respectively, and a mean distance of 1.18 ± 0.36 mm [4]. Their values were generally larger than those determined here, as shown in Table 1. Differences in the image thickness could explain these deviations. That is, in their study, the CT image thickness was 2 or 3 mm, which is larger than the 1.25-mm thickness used in this study. Additionally, their MR image thickness was 1 mm, which is smaller than the 1.5-mm thickness used in this study. Additionally, the box phantom might have provided less information for co-registration procedures than the anthropomorphic phantom. Nakazawa et al. obtained co-registration errors between stereotactic CT and co-registered MR images using two box phantoms and an anthropomorphic phantom [5]. However, their results could not be directly compared with the present results because they provided only the maximum relative errors in a plane or along an axis. By combining the results determined using box and anthropomorphic phantoms in their study, the mean maximum three-dimensional error was estimated to be 2.1 ± 0.3 mm, which is similar to the results found in this study, as shown in Fig 9B. Those authors also showed that the mean three-dimensional displacement of the center of gravity of the target was 1.06 ± 0.60 mm based on eleven vestibular schwannoma cases. Their results were consistent with the mean displacement of the AC and PC data found here: 1.0 ± 0.3 mm.

Li et al. reported that the mean one-dimensional deviations between frame-based CT images and co-registered CBCT images of twenty patients were −0.19 ± 0.32 mm, 0.08 ± 0.29 mm, and −0.35 ± 0.50 mm along the x-, y-, and z-axes, respectively. The mean three-dimensional displacement was 0.40 ± 0.66 mm [7]. Their CBCT was a research prototype with an image voxel size of 1 mm, which is twice as thick than used in the current study. Their results were larger than the deviations found using the no-movement setup in this work and were similar to the deviations in the arbitrary-movement setup shown in Fig 8. Li et al. also reported the intrafraction patient movements observed by co-registering the CBCT images of seventeen patients with reference CBCT images. The mean one-dimensional differences were −0.03 ± 0.05 mm, −0.03 ± 0.18 mm, and −0.03 ± 0.12 mm for the x-, y-, and z-axes, respectively. The mean three-dimensional displacement was 0.05 ± 0.22 mm. Because patients were fixed with a frame, their setups corresponded to the no-movement setup of CBCT/CBCT co-registration, and the results were consistent with the results of this study, as shown in Fig 6. Interestingly, their mean three-dimensional deviation was smaller than that determined here (0.2 ± 0.1 mm), although they used patient images with a mean interval of 82 minutes between CBCT images.

All previous studies on co-registration accuracy used a fiducial marker-based coordinate system as their ‘gold standard’ reference system. In this study, however, the CBCT image series taken with CTDI_w_ = 6.3 mGy was used as the reference system because the mean maximum deviation of the CBCT images over a one-year period was 0.07 ± 0.02 mm; in contrast, the mean fiducial marker position error of the stereotactic CT images was 0.3 mm, and the mean maximum error was 0.8 mm.

Regarding the effects of the ROC on accuracy, the general expectation was that the ‘Mid’ range would provide the minimum error because MR distortion errors should be smaller in the central area [3,10]. In this study, the mean MR distortions obtained using the CIRS 603A phantom MR images were 0.6 ± 0.2 mm, 0.4 ± 0.2 mm, and 0.6 ± 0.2 mm in the ‘Low’, ‘Mid’, and ‘High’ regions, respectively. The fact that the minimum co-registration error was associated with the ‘Low’ region could be explained because mainly bony structures were used for co-registration with CBCT images because of the low soft tissue contrast of the GK Icon CBCT. Thus, the increased mutual information provided by the complex bony structure in the base of the skull was presumed to be more important than MR image distortion in the co-registration procedure. Pappas EP et al. reported that the mean distortion error in MR images used for GKSRS was 0.95 mm but that it decreased to 0.60 mm when only points within 7 cm of the coordinate center were included and to 0.47 mm when this radius was decreased to 5 cm [3]. In this study, the ‘Low’ region was more than 1 cm higher than the frame in all cases, and as a result, most of the points in the ‘Low’ region were within 6 cm of the center. The inclusion of other areas located above the skull base area did not improve the co-registration accuracy, likely because few bony structures in this area could be used in mutual information processing, and the effects from image distortion accumulated. Although the effects on target coverage and minimum dose were not statistically significant, using the ‘Low’ region appears to be more desirable than using the ‘Large’ region.

The most common image series used in GKSRSs are MR images taken with a stereotactic frame. The mean MR image distortion errors, which are important for the accuracy of frame-based GKSRS, were shown to be between 0.53 and 0.60 mm in a phantom study [3]. The maximum distortions ranged from 1.41 to 1.64 mm within 7 cm of the coordinate center. In the present study, the mean value of the maximum shot displacement after co-registration of patient stereotactic MR images taken with a frame was 1.0 ± 0.5 mm using the ‘Large’ ROC, whereas the displacement of the AC and PC decreased to 0.8 ± 0.3 mm with the ‘Low’ region. Neumann J.O. et al. also reported that the errors from the spatial distortion of MR images were reduced after co-registering MR images with stereotactic CT images [10]. These errors from image co-registration may be even smaller in practical cases because patient MR images are taken without a frame for frameless GKSRS. When no frame was used, the mean and maximum distortions of MR images were reported to decrease to 0.48 mm and 0.98 mm, respectively [3]. Thus, their effects on co-registration error should also be reduced.

The effects of image co-registration errors of LGP in a single shot plan are different from those in a multiple shot plan. In a single shot plan, the image co-registration error is directly reflected in the translation and/or rotation of the dose distribution, though the irradiated volume is preserved. Translation along each axis and rotations around the x- and y-axes cause corresponding translation and rotation of the dose distribution. Rotation around the z-axis has no effect because a cylindrical symmetry exists around the z-axis in the case of single shot dose distribution. In a multiple shot plan, the effects of some shot movements could cancel each other out, and the net effect on the dose distribution becomes smaller such that the global measures are less affected [11].

## Conclusions

The co-registration of GK Icon CBCT images, which is used to verify the patient setup, resulted in mean deviations of 0.2 mm. The stereotactic coordinate assignment based on the co-registration of patient stereotactic MR images with GK Icon CBCT images had sub-millimeter deviations, and the accuracy was similar to those of the MR images used in frame-based GKSRS. To reduce the co-registration error between stereotactic MR images and CBCT images, the lower part of the skull, including the base of the skull but excluding the lowest movable part, is recommended as the ROC.

## Supporting information

S1 Table The frequency counts after CBCT to CBCT co-registration. Frequency counts of one- and three-dimensional deviations after co-registration between the setup CBCT images and reference CBCT images.
(PDF)

S2 Table The frequency counts after CT to CBCT co-registration. Frequency counts of one- and three-dimensional deviations after co-registration of clinical serial CT images and CBCT images.
(PDF)
