# Supplementary material for: Assessment of image co-registration accuracy for frameless gamma knife surgery
Source: PLoS One. 2018 Mar 2;13(3):e0193809. doi: 10.1371/journal.pone.0193809 (PMC5834193; doi:10.1371/journal.pone.0193809)
Supplement: S1 Table — Frequency counts of one- and three-dimensional deviations after co-registration between the setup CBCT images and reference CBCT images. (PDF) [file pone.0193809.s001.pdf]

S1 Table. Frequency Counts for CBCT-CBCT co-registration

Before Coregistration					
Deviation	Counts_X	Counts_Y	Counts_Z	3D_deviation	Counts
-0.275	0	0	0	0.025	4
-0.225	0	1	0	0.075	18
-0.175	4	2	1	0.125	18
-0.125	7	5	2	0.175	16
-0.075	11	13	7	0.225	2
-0.025	9	12	12	0.275	1
0.025	19	13	25	0.325	1
0.075	3	7	6	0.375	0
0.125	5	7	7	0.425	0
0.175	2	0	0	0.475	0
0.225	0	0	0	0.525	0
0.275	0	0	0		
0.325	0	0	0		
0.375	0	0	0		

Co-registration without movement					
Deviation	Counts_X	Counts_Y	Counts_Z	3D_deviation	Counts
-0.275	0	0	0	0.025	1
-0.225	0	0	2	0.075	14
-0.175	0	0	5	0.125	22
-0.125	8	3	3	0.175	20
-0.075	9	14	2	0.225	12
-0.025	16	16	7	0.275	4
0.025	11	18	16	0.325	2
0.075	18	7	18	0.375	0
0.125	10	11	9	0.425	0
0.175	2	6	9	0.475	0
0.225	0	0	3	0.525	0
0.275	0	0	0		
0.325	1	0	1		
0.375	0	0	0		

Co-registration after rotation only					
Deviation	Counts_X	Counts_Y	Counts_Z	3D_deviation	Counts
-0.275	0	1	0	0.05	3
-0.225	3	2	1	0.1	20
-0.175	3	7	2	0.15	52
-0.125	7	11	22	0.2	35
-0.075	38	20	24	0.25	18
-0.025	25	29	37	0.3	3
0.025	47	35	42	0.35	2
0.075	30	34	25	0.4	2
0.125	37	23	18	0.45	0
0.175	16	11	4	0.5	0
0.225	8	7	4	0.55	0
0.275	2	0	1		
0.325	0	0	0		
0.375	0	0	0		

Co-registration after arbitrary movement					
Deviation	Counts_X	Counts_Y	Counts_Z	3D_deviation	Counts
-0.375	0	0	1	0.15	13
-0.325	0	1	0	0.2	6
-0.275	0	4	1	0.25	19
-0.225	5	7	0	0.3	8
-0.175	11	8	2	0.35	11
-0.125	9	11	7	0.4	3
-0.075	15	14	6	0.45	1
-0.025	8	3	7	0.5	0
0.025	8	4	6	0.55	0
0.075	4	6	15		
0.125	5	4	4		
0.175	1	4	6		
0.225	0	0	6		
0.275	0	0	4		
