# Supplementary material for: Assessment of image co-registration accuracy for frameless gamma knife surgery
Source: PLoS One. 2018 Mar 2;13(3):e0193809. doi: 10.1371/journal.pone.0193809 (PMC5834193; doi:10.1371/journal.pone.0193809)
Supplement: S2 Table — Frequency counts of one- and three-dimensional deviations after co-registration of clinical serial CT images and CBCT images. (PDF) [file pone.0193809.s002.pdf]

S2 Table. Frequency Counts for CT-CBCT co-registration

Before Coregistration					
Deviation	Counts_X	Counts_Y	Counts_Z	3D_deviation	Counts
-0.8	0	0	0	0.05	0
-0.6	0	0	0	0.15	2
-0.4	4	2	3	0.25	4
-0.2	5	6	5	0.35	7
0	3	18	13	0.45	4
0.2	5	4	6	0.55	5
0.4	6	0	3	0.65	4
0.6	5	0	0	0.75	4
0.8	2	0	0	0.85	0
				0.95	0

Co-registration wihout movement					
Deviation	Counts_X	Counts_Y	Counts_Z	3D_deviation	Counts
-0.8	0	0	0	0.05	0
-0.6	0	0	2	0.15	3
-0.4	0	0	3	0.25	13
-0.2	3	4	6	0.35	5
0	16	13	4	0.45	4
0.2	10	13	9	0.55	4
0.4	1	0	4	0.65	1
0.6	0	0	2	0.75	0
0.8	0	0	0	0.85	0
				0.95	0

Co-registration after arbitrary movement					
Deviation	Counts_X	Counts_Y	Counts_Z	3D_deviation	Counts
-0.8	1	0	0	0.05	4
-0.6	3	3	3	0.15	6
-0.4	12	20	9	0.25	8
-0.2	31	35	19	0.35	11
0	24	21	15	0.45	15
0.2	9	2	8	0.55	15
0.4	2	1	22	0.65	17
0.6	0	0	6	0.75	4
0.8	0	0	0	0.85	1
				0.95	1
